# Pandemic Vaccine Preparedness—Have We Left Something Behind?

**DOI:** 10.1371/journal.ppat.1000482

**Published:** 2009-06-26

**Authors:** Ilaria Capua, Anna Kajaste-Rudnitski, Elena Bertoli, Elisa Vicenzi

**Affiliations:** 1 Istituto Zooprofilattico Sperimentale delle Venezie, OIE/FAO and National Reference Laboratory for Newcastle Disease and Avian Influenza, OIE Collaborating Centre for Epidemiology, Training and Control of Emerging Avian Diseases, Legnaro, Padova, Italy; 2 Viral Pathogens and Biosafety Unit, Division of Immunology, Transplantation and Infectious Diseases, San Raffaele Scientific Institute, Milan, Italy; The Fox Chase Cancer Center, United States of America

Influenza A viruses all originate from aquatic birds, which are their natural reservoir. From this vast, ever-present and global source they are able to cross the species barrier and infect a variety of hosts, including humans. Progressive viral adaptation of avian-origin viruses to novel hosts including domesticated birds, pigs, horses, dogs, or humans may result in widespread viral circulation and in the establishment of endemic viruses in a given population. In humans, influenza A infections usually are caused by endemic seasonal viruses and much less frequently by animal influenza viruses that cross the species barrier. A few times each century, some of the animal viruses also gain the capacity to sustain transmission among human populations, resulting in a pandemic. By definition, any emerging pandemic virus will be different antigenically from both human vaccine virus strains and contemporary human viruses, and so the human population will be immunologically naïve to a significant degree to the new virus before it spreads widely. To date, only viruses of the H1, H2, and H3 subtypes are known to have caused pandemics and establish subsequent global circulation.

It has been shown that all pandemic viruses emerging in the 20th century have had an avian influenza progenitor virus donating at least the haemagglutinin gene [1]. Since 1997, most scientific studies have focused on avian H5, and to a minor extent avian H7 and H9 subtype viruses, because these viruses have caused repeated zoonotic human infections and have spread widely in poultry over the past 10 years or more. So far, animal H1, H2, and H3 subtype viruses have been excluded from international research aiming at the development of pandemic vaccine candidates. The rationale for this is that the human population is considered sufficiently immune to H1 and H3 viruses due to exposure or vaccination against seasonal influenza viruses. In addition, the population over 40 years of age is largely immune to the H2 subtype viruses that circulated between 1957 and 1968.

There are significant antigenic differences within subtypes that change over time as these viruses evolve, and this requires a semi-annual review and frequent update of vaccine strain candidates for human seasonal influenza vaccines. The degree of relatedness between the strains contained in the seasonal vaccine and the drifted strains that are isolated the following year is assessed through cross haemagglutination inhibition (HI) tests. If the results of these tests suggest that cross HI levels are low, the vaccine is updated to include the most recent strains.

We reasoned that if a semi-annual review of the antigenic characteristics of human H1 and H3 viruses is necessary due to antigenic drift resulting from immunologic pressure, it was possible that contemporary avian H1 and H3 viruses would not be cross-reactive with contemporary seasonal influenza A viruses.

We tested by HI 30 human serum specimens, obtained 3–5 weeks after the administration of the 2006–2007 seasonal vaccine, against A/Wisconsin/67/2005/H3N2, A/New Caledonia/20/99/H1N1, and A/Singapore/57/H2N2 to establish serological titres to human influenza viruses. We then used a selection of contemporary avian viruses, namely A/mallard/Italy/432-21/08 H1N1; A/shoveler/Italy/6965-6/07 H1N3; A/duck/China/626-2/07 H1N8; A/duck/Italy/3459/06 H2N2; A/mallard/Italy/6512-69/07 H2N6; A/duck/Italy/3139-1/06 H3N2; and A/duck/Italy/6207/08 H3N6 as test antigens in the HI test using both chicken and equine red blood cells [2],[3]. We subsequently selected the most high titred sera and performed a serum neutralisation assay in SPF embryonated fowl's eggs [4].

We found that HI antibodies present in human sera, induced by vaccination using the seasonal 2006–2007 vaccine, do not significantly cross-react with contemporary Eurasian lineage avian influenza viruses of the H1 or H3 subtypes. These results are confirmed by the neutralisation assay. In contrast, antibodies in some individuals over 40 years of age against A/Singapore/57/H2N2 are cross-reactive both in HI and serum neutralisation assays with contemporary avian H2 viruses. Results are presented in Table 1.

**Table 1:** HI and SN Titres in Human Sera against Human and Avian Viruses of the H1, H2, and H3 Subtype.

Age of Patient	Human Viruses			Avian Viruses													
	H1N1*	H3N2**	H2N2***	H1N1		H1N3		H1N8		H2N2		H2N6		H3N2		H3N6	
				A/mallard/Italy/432-21/08		A/shoveler/Italy/6965-6/07		A/duck/China/6262/07		A/duck/Italy/3459/06		A/mallard/Italy/651269/07		A/duck/Italy/31391/06		A/duck/Italy/6207/8	
	HI	HI	HI	HI	SN	HI	SN	HI	SN	HI	SN	HI	SN	HI	SN	HI	SN
35	128∧	128	-° (-°°)	- (-)	•	- (-)	•	- (-)	•	- (-)	•	- (•)	•	- (-)	•	- (-)	•
49	64	128	64 (-)	- (-)	•	- (-)	•	- (-)	•	- (-)	•	- (•)	•	- (-)	•	- (-)	•
51	128	64	256 (64)	- (-)	•	- (-)	•	- (-)	•	- (-)	•	- (•)	•	- (-)	•	- (-)	•
41	128	128	256 (128)	- (-)	•	- (-)	•	- (-)	•	32(-)	≥1∶40	32(•)	-	- (-)	•	32 (-)	-
31	64	64	- (-)	- (-)	•	- (-)	•	- (-)	•	- (-)	•	- (•)	•	- (-)	•	- (-)	•
48	128	128	32 (-)	- (-)	•	- (-)	•	- (-)	•	- (-)	•	- (•)	•	- (-)	•	- (-)	•
56	128	128	512 (32)	- (-)	•	- (-)	•	- (-)	•	64 (-)	≥1∶40	64(•)	≥1∶40	- (-)	•	- (-)	•
32	128	64	- (-)	- (-)	•	- (-)	•	- (-)	•	- (-)	•	- (•)	•	- (-)	•	- (-)	•
58	64	64	128 (-)	- (-)	•	- (-)	•	- (-)	•	- (-)	•	- (•)	•	- (-)	•	- (-)	•
41	128	128	- (-)	- (-)	•	- (-)	•	- (-)	•	- (-)	•	- (•)	•	- (-)	•	- (-)	•
47	512	256	256 (256)	- (-)	-	- (-)	-	- (-)	-	64 (128)	-	32(•)	≥1∶40	- (-)	-	1024 (1024)	-
22	256	256	- (-)	- (-)	-	- (-)	-	- (-)	-	- (-)	-	- (•)	-	- (-)	-	- (-)	•
36	512	256	- (-)	- (-)	-	- (-)	-	- (-)	-	- (-)	-	- (•)	-	- (-)	-	32 (-)	-
48	256	256	512 (128)	- (-)	-	- (-)	-	- (-)	-	64 (-)	-	64(•)	-	- (-)	-	- (-)	-
50	256	256	512 (256)	- (-)	-	- (-)	-	- (-)	-	32(64)	≥1∶40	32(•)	≥1∶40	- (-)	1∶20	- (-)	-
47	512	256	128 (128)	- (-)	-	- (-)	-	- (-)	-	32(-)	-	- (•)	-	- (-)	-	- (-)	-
44	512	256	128 (32)	- (-)	-	- (-)	-	- (-)	-	32 (-)	-	32(•)	-	- (-)	-	32 (-)	-
50	512	256	256 (128)	- (-)	-	- (-)	-	- (-)	-	32(32)	≥1∶40	32(•)	≥1∶40	- (-)	1∶20	- (-)	-
53	256	512	32 (-)	- (-)	-	- (-)	-	- (-)	-	- (-)	•	- (•)	•	- (-)	-	32 (-)	-
20	512	256	- (-)	- (-)	-	- (-)	-	- (-)	-	- (-)	•	- (•)	•	- (-)	-	- (-)	-
52	8	32	128 (128)	- (-)	•	- (-)	•	- (-)	•	- (-)	•	- (•)	•	- (-)	•	- (-)	•
47	64	64	32 (-)	- (-)	•	- (-)	•	- (-)	•	- (-)	•	- (•)	•	- (-)	•	- (-)	•
22	64	32	- (-)	- (-)	•	- (-)	•	- (-)	•	- (-)	•	- (•)	•	- (-)	•	- (-)	•
29	64	64	- (-)	- (-)	•	- (-)	•	- (-)	•	- (-)	•	- (•)	•	- (-)	•	- (-)	•
35	32	64	- (-)	- (-)	•	- (-)	•	- (-)	•	- (-)	•	- (•)	•	- (-)	•	- (-)	•
40	64	64	- (-)	- (-)	•	- (-)	•	- (-)	•	- (-)	•	- (•)	•	- (-)	•	- (-)	•
43	32	32	- (-)	- (-)	•	- (-)	•	- (-)	•	- (-)	•	- (•)	•	- (-)	•	64 (-)	-
51	32	64	256 (128)	- (-)	•	- (-)	•	- (-)	•	- (-)	•	32(•)	•	- (-)	•	- (-)	•
57	32	64	128 (-)	- (-)	•	- (-)	•	- (-)	•	- (-)	•	- (•)	•	- (-)	•	- (-)	•
27	32	32	- (-)	- (-)	•	- (-)	•	- (-)	•	- (-)	•	- (•)	•	- (-)	•	- (-)	•

*: A/Wisconsin/67/2005/H3N2.

**: A/New Caledonia/20/99/H1N1.

***: A/Singapore/57/H2N2.

- = ≤1∶16 for HI, <1∶20 for SN.

∧ = Titres expressed as the highest dilution at which full haemagglutination inhibition is observed.

° = 0.5% chicken RBC solution.

°° = 1% horse RBC solution.

• = Not done.

The results of this preliminary investigation show that most of the human serum specimens tested, regardless of their titre against H1 and H3 human viruses, were negative or exhibited only low antibody titres against avian viruses of the same subtype.

Interestingly, antibodies against A/Singapore/57/H2N2, present in most samples collected from patients over 40 years of age exhibited a higher degree of cross-reactivity with avian viruses of the same subtype. It could be speculated that this virus has maintained a greater antigenic component of its avian progenitor since it has only circulated in the human population for 11 years (1957–1968) compared to H3N2 for 41 years (1968–present).

These data show that post-seasonal influenza vaccination antibodies do not appear to be cross-reactive with contemporary avian H1 or H3 subtype viruses of Eurasian lineage and suggest that an avian influenza virus of the H1 or H3 subtype could again donate the haemagglutinin gene and form part of the next pandemic virus.

While H2 subtype viruses are already perceived as a potential threat, due to the fact that the population under 40 years old is immunologically naïve to H2 viruses, the possible emergence of a human pandemic virus with an avian H1 or H3 haemagglutinin has not been discussed to any great extent. It is likely that our preliminary findings reflect the existence of a population of animal viruses of the H1 and H3 subtype that are very distant antigenically and genetically from human influenza seasonal strains and thus could ignite a pandemic. Evidence of this risk has been reported following cases of swine H1N1 and H3N2 infection in humans [5],[6]. Recently, sustained human-to-human transmission of a swine influenza virus of the H1N1 subtype across the North American continent has disseminated the virus to Europe, Asia, South America, and Oceania [7]. If this novel virus continues to spread and becomes established fully in the human population, a specific vaccine will be required. The development of any such vaccine would have benefited from the generation of knowledge prior to the outbreak, as recommended for H5N1.

Although many of the public health and clinical preparations for a pandemic are independent of the actual virus, certain preparations such as the development and production of a human pandemic vaccine system depend greatly on the specific virus. Little is known about the antigenicity and in vitro growth kinetics of animal H1, H2, and H3 viruses since they have been of scarce interest to the veterinary community, as they only cause mild disease in animals and are not notifiable infections. However, a significant number of such virus isolates are already available for antigenic and genetic characterisation in veterinary laboratories worldwide, and it would seem prudent for medical and veterinary virologists to work together to investigate the cross-reactivity and pandemic potential of animal H1, H2, and H3 subtypes along with other recognized potential pandemic subtypes such as H5, H7, and H9. Such work could build essential bridges between the public health and veterinary sectors and improve pandemic preparedness efforts globally.

*Preliminary data on this investigation was presented at the closed OIE/FAO/WHO Tripartite meeting in Paris, 3–4 February 2009*.
